# A side-by-side comparison of variant function measurements using deep mutational scanning and base editing

**DOI:** 10.1093/nar/gkaf738

**Published:** 2025-07-31

**Authors:** Ivan Sokirniy, Haider Inam, Marta Tomaszkiewicz, Joshua Reynolds, David McCandlish, Justin Pritchard

**Affiliations:** Huck Institute for the Life Sciences, University Park, PA 16802, United States; Huck Institute for the Life Sciences, University Park, PA 16802, United States; Department of Biomedical Engineering, University Park, PA 16802, United States; Huck Institute for the Life Sciences, University Park, PA 16802, United States; Department of Biomedical Engineering, University Park, PA 16802, United States; Huck Institute for the Life Sciences, University Park, PA 16802, United States; Department of Biomedical Engineering, University Park, PA 16802, United States; Simons Center for Quantitative Biology, Cold Spring Harbor Laboratory, Cold Spring Harbor, NY 11724, United States; Huck Institute for the Life Sciences, University Park, PA 16802, United States; Department of Biomedical Engineering, University Park, PA 16802, United States

## Abstract

Variant annotation is a crucial objective in mammalian functional genomics. Deep mutational scanning (DMS) using saturation libraries of complementary DNAs (cDNAs) is a well-established method for annotating human gene variants, but CRISPR base editing (BE) is emerging as an alternative. However, questions remain about how well high-throughput BE measurements can annotate variant function and the extent of downstream experimental validation required. This study is the first direct comparison of cDNA DMS and BE in the same lab and cell line. We focus on how well short guide RNA (sgRNA) depletion or enrichment is explained by the predicted edits within the editing “window” defined by the sgRNA. The most likely predicted edits enhance the agreement between a “gold standard” DMS dataset and a BE screen. A simple filter for sgRNAs making single edits in their window could sufficiently annotate a large proportion of variants directly from sgRNA sequencing of large pools. When multi-edit guides are unavoidable, directly measuring edits in medium-sized validation pools can recover high-quality variant annotation data. Our data show a surprisingly high degree of correlation between base editor data and gold standard DMS. We suggest that the main variable measured in base editor screens is the desired base edits.

## Graphical Abstract

**Graphical Abstract:**
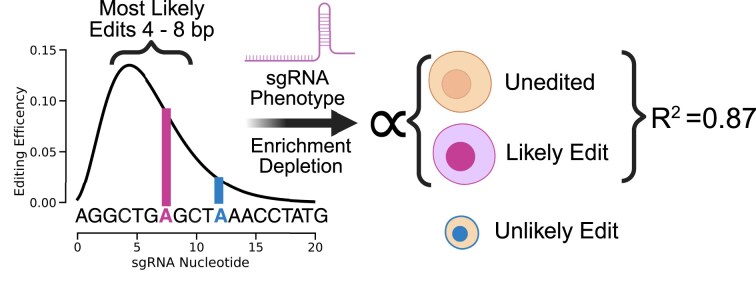

## Introduction

A major goal of mammalian functional genomics is to understand the genotype-phenotype relationship [1]. However, achieving this lofty goal requires large-scale experiments that can, in parallel, examine both genotypes and phenotypes in pooled assays. While gene-level loss-of-function screens in mammalian cells rose to prominence with the invention of RNAi-based screening tools [2–4] in the early 2000s, and eventually CRISPR/CRISPRi [5–8] in 2012–2013, the high throughput annotation of individual coding variants in mammalian cell lines is a more recent innovation [9, 10].

High-throughput variant annotation studies in mammalian cell systems include CRISPR-mediated HDR, cDNA library introduction, base editing (BE) by cytosine or adenosine base editors, and prime editing. For the purposes of this introduction, we have included Supplementary Table S1 highlighting the strengths and weaknesses of these approaches alongside key references from the present literature. Importantly, HDR is a powerful tool but it has not been widely adopted outside of haploid HAP1 cells [11–15]. cDNA libraries can be introduced by viral vectors into virtually any mammalian cell line or incorporated into landing pads [16–18] in some cell lines and are easy to synthesize externally, but the artificial expression context is a source of concern. BE is becoming a remarkably efficient way to edit variants in the endogenous genomic context [19, 20], libraries of sgRNAs are easy to clone [21], and moving between cell lines is well demonstrated [22], but it suffers from a limited mutational repertoire and the experimental complexity of potentially making multiple mutations in a single editing window. Prime editing is becoming much more efficient [23], but it still requires MLH1 deficiency [24, 25] and requires pre-identification of strong epegRNA sequences for highest efficiency [23, 26]. Thus, at present, the two methods that are demonstrated to be the most usable across many wild type (WT) mammalian cell lines are cDNA libraries and base editor libraries. As mammalian geneticists and cell biologists work in over 1000 different cell lines, we focused this paper on the most portable technologies that have been shown by others to allow for the measurement of variant function in many different cell lines with little cell-type-specific modifications. Because of this, we have chosen to compare BE to cDNA-based deep mutational scanning (DMS).

DMS is a leading technology in the field of high-throughput variant annotation [9, 10]. DMS libraries can involve heterologous expression of large cDNA libraries of single amino acid mutations that encompass all 20 possible amino acids at every position. When performed in mammalian cell lines, these libraries are introduced via a transduction [27–29] or into a safe harbor “landing pad” [16–18]. While DMS is capable of providing comprehensive measurements of variant effects [10, 30, 31], DMS has nonetheless been difficult to scale to large genes or to multi-gene families, and the measurements may not reflect the effects of the same mutations at the endogenous genomic locus. Moreover, the technical challenges involved in DMS can lead to variable dataset quality [32].

As a functional genomic technology, BE is at development stage. BE screens use nCas9 to target a deaminase to a specific site in the genome and generate transition mutations (C > T for cytosine base editors—(CBEs) or A > G for adenine base editors—(ABEs) [19, 20]. BE screens use a surrogate measure of genotype by sequencing the short guide RNA (sgRNA) sequence, which allows BE screens to measure phenotypes across the genome [33–35]. Moreover, base editor screens have other major advantages that can include the ability to edit at the endogenous genomic locus and the ability to identify splicing defects [33, 36–39]. However, base editor screens also present certain challenges. The primary challenges are (i) BE efficiency (only a portion of individual cells harboring an sgRNA are likely to be edited, and some cell lines do not edit well [40]); (ii) off-target editing (while editing is largely constrained to a small window within the sgRNA non-target sequence, some off-target editing is likely occurring [41, 42]); (iii) bystander editing (when more than one possible edit occurs in an on-target editing window, the amino acid variant(s) made are more challenging to infer [43, 44]); and (iv) protospacer adjacent motif (PAM) requirements (PAMs limit where sgRNAs target [45], and current PAM-less Cas9 variants appear to have decreased efficiency [46–48]). These issues have led the field to view BE screens as a method for initial identification of interesting variants and regions but with limited capability to directly annotate loss-of-function phenotypes.

When competing high-throughput measurement methodologies can generate similar data, it can be extremely useful to directly benchmark these methods against each other. For instance, the direct comparison of CRISPR Cas9 LOF screens and RNAi screens suggested that CRISPR is a more sensitive and specific technique for identifying essential LOF phenotypes, but that RNAi screens can help understand the dosage sensitivities of essential genes and can sometimes rescue false negatives in CRISPR screens for a subset of biological functions [49–51]. Additionally, the direct comparisons of high throughput drug sensitivity measurements found that the precise metrics and methods that are used in comparing datasets can create different conclusions on dataset reliability and usability. Together, these high-profile efforts highlight the importance of a careful comparison of high throughput datasets using multiple metrics and the public dissemination of the resultant data [52, 53].

Here we perform the first direct comparison between BE and DMS in the same cell line in the same lab. To accomplish this, we use the Ba/F3 cell system. This allows for a direct comparison and eliminates differences in genetic context as a confounding variable driving the differences in measurements between the approaches. Using this system, we identify specific data filters that generate largely matching conclusions about the phenotypes of loss-of-function variants. Furthermore, we demonstrate that applying these data filters enhances the correlation within the endogenous genomic context, indicating their robustness across different cell models. We also identify a two-step high throughput workflow for base editor screens that can streamline the validation of variant interpretation in pools by directly sequencing the edited variant fraction with error corrected sequencing [54, 55].

## Materials and methods

### DMS library preparation and screen

BCR-ABL cDNA was cloned downstream of EGFP in the pUltra (Addgene #24129) lentiviral vector by GenScript to make pUltra BCR-ABL WT (Addgene #210432). Twist Bioscience generated a saturating mutagenesis library of single amino acid changes in the N-lobe of the ABL kinase domain. NEB Stable chemically competent (NEB #C3040I) cells were transformed with the SM library, with a coverage of >1000×, onto 15-cm LB agar plates with ampicillin. After 48 h at 30°C, colonies were scrapped off the agar and plasmid DNA was extracted using Omega Bio-Tek E.Z.N.A. Plasmid DNA Midi kit (#D6904). HEK293Ts were transfected with 35 μg of saturating mutagenesis ABL library and 10 μg of helper plasmids (1:1:1:1) in 10-cm dishes using Thermo Fisher Lipofectamine 3000 (5 Lipo : 1 DNA). The next day, the media was changed to fresh RPMI (Cytivia SH30027.02). After 36 h, viral RPMI media was used to infect Ba/F3s, in the presence of 1 μg/ml mouse IL-3 (peprotech 213-13) and 6 μg/ml polybrene, at a low multiplicity of infection. After another 36 h, Ba/F3s were maintained in RMPI with 1 ng/ml IL-3. Infected cells were enriched by fluorescence-activated cell sorting (FACS) on EGFP at the Penn State Flow Cytometry Core facility.

At the start of the DMS screen, IL-3 was removed, and 30 million cells were saved to establish a baseline mutation frequency. Approximately 5 million cells were treated with DMSO for 6 days. Media was refreshed on day 3. Cell count was tracked by a BD Accuri C6 Plus flow cytometer. Cells were maintained in exponential phase. If cell viability was <90%, then viable cells were enriched by Ficol-Paque (Cytiva).

### DMS library preparation and single-strand consensus sequencing

High-quality genomic DNA was extracted by Monarch Genomic DNA Purification Kit (NEB #T3010S). Then, a modified and scaled-up CRISPR-DS workflow was used to determine accurate variant distributions [56]. Equimolar crRNAs (IDT, 5′-caagtgggagatggaacgca-3′, 5′-catgacctacgggaacctcc-3′) were pooled and combined with tracrRNA (IDT) (final concentration: 10 μM each). Guide RNA (gRNA) duplexes were formed by heating to 95°C for 5 min, followed by cooling to room temperature for 5 min. RNPs were assembled by incubating 10 μl of gRNA duplexes with 1.6 μl HiFi Cas9 (IDT), 3 μl 1× CutSmart buffer (NEB), and 15.4 μl nuclease-free water for 20 min at room temperature. Genomic DNA (20 μg, resuspended in 1× CutSmart buffer) was digested by adding 20 μl of pre-assembled RNPs and incubating at 37°C for 1 h. Proteinase K (10 μl, 20 mg/ml, NEB) was then added, and the sample incubated at 56°C for 10 min. Undigested high-molecular-weight DNA was removed using 0.5× AMPure XP beads. A second purification with 1.8× AMPure XP beads was performed to remove short fragments, and DNA was eluted in 30 μl TE. DNA was quantified using a Qubit fluorometer. A-tailing, unimolecular identifier (UMI) ligation, hybridization capture with custom probes, and PCR amplification were performed as described previously [57, 58].

One-hundred-fifty-nucleotide paired-end sequencing of the UMIs and mutagenized region was done on an Illumina NovaSeq 6000. For each sample, Du Novo [59] was used to generate error-corrected single-strand consensus from the UMI barcodes. Then bwa-mem2 was used to align the census to human ABL cDNA. After filtering out for mouse ABL reads, aligned reads with less than five mismatches would undergo variant calling and annotation using a custom R script. Briefly, for each alignment, variants were converted from the MDZ read tag. Mutant growth rates were calculated using exponential growth equation and the mutant allele frequency (MAF):


(1) \begin{eqnarray*}
{\rm growth}\;{\rm rate} = \ln \left( {\frac{{{\rm MA{F_1}} \times {\rm Coun{t_1}}}}{{{\rm MA{F_0}} \times {\rm Coun{t_0}}}}} \right) \div \left( {{\rm Tim{e_1}} - {\rm Tim{e_0}}} \right)
\end{eqnarray*}

where the subscripts 0 and 1 denote the initial and final time point, respectively. Cell counts and the splitting ratio were used to account for dilution due to cell splitting during the DMS screen. Time is measured in units of hours. Skewed Gaussian mixture models were fit over the bimodal distribution of mutant growth rates using the Curve_fit function from the Scipy Python package [60]. Z-score cutoff for DMS data was determined by fit mean and standard deviation of the WT like component of the mixture distribution.

### Base editor library preparation, screen, and sequencing

BCR-ABL tiling gRNA sequences were generated by CHOP-CHOP [61] with “NGN” PAM setting. Guides were cloned into lenti-sgRNA hygro vector (Addgene #104991) by GenScript to make the BCR-ABL sgRNA library. ABE8e SpG plasmid was made by deleting the U6 sgRNA cassette from pRDA_479 (Addgene #179099) [62] using NEB KLD (NEB #M0554S). For the CBEd SpG plasmid, a highly efficient and specific CBE from TadCBEd (Addgene #193835) [63] was subcloned to replace the ABE. The nickase (nSpG) plasmid was created by deleting the editor by NEB KLD.

Ba/F3s infected with pUltra BCR-ABL WT (Addgene #210432) were allowed to grow in the absence of IL-3. After 5 days >95% of the Ba/F3s were EGFP+. Then they were infected with either ABE8e SpG (Addgene #235044), CBEd SpG (Addgene #235045), or nSpG (Addgene #235046) and selected with 1 μg/ml puromycin for 5 days. K562 ABE8e SpG were created similarly. In a 15-cm dish HEK293Ts were transfected with 60 μg of the BCR-ABL and control sgRNA libraries and 40 μg of helper plasmids (1:1:1:1) using calcium phosphate. The next day DMEM media was changed to RPMI. Three independent infections of Ba/F3 pUltra BCR-ABL WT ABE8e cells were performed at low multiplicity of infection and >500× coverage. CBEd SpG and nSpG carrying cells were infected similarly. Transfected HEK23Ts were saved to establish a baseline sgRNA frequency. Three days after sgRNA library infection, the cells were selected with 1 mg/ml hygromycin for 6 days and pelleted.

The K562 screen largely mirrored the Ba/F3 BCR-ABL screen, with both screens lasting 9 days. K562 cells were initially infected with ABE8e SpG and selected with 1 μg/ml puromycin for 5 days, consistent with the Ba/F3 protocol. Subsequently, these K562 ABE8e SpG cells were infected with the BCR-ABL library, also as described previously, but selected with 200 μg/ml hygromycin for 6 days instead. For the comparison between the K562 and Ba/F3 screens, we used a frequency cutoff of 1:10 000 to identify high-confidence sgRNAs.

Genomic DNA was extracted using phenol-chloroform [64] and quantified by Qubit. Staggered PCR was used to extract sgRNA sequences from 10 μg of genomic DNA, as described previously [21], with two modifications: custom forward staggered primers were used, and PCR amplicons were gel extracted using Omega Bio-Tek Gel Extraction kit (#D6294).

### sgRNA analysis

gRNA amplicons were trimmed with CutAdapt [65] to remove the U6 promoter and gRNA scaffold. The remaining sequences were aligned to a reference list of guides using Bowtie [66]. Guide counts were established by Counter from the collections Python package. pyDESeq2 [67] was used to determine the fold change in guides between time points. The fold change was converted to growth rates using Equation 1 above assuming WT growth rate. Z-score cutoff for ABE data was based on the human targeting (AAVS1, CCR5, and ROSA26) negative control sgRNA.

### Verification screen, sequencing, and analysis

After making three independent Ba/F3 BCR-ABL WT ABE8e cell lines as above, IL-3 was returned to the RPMI media. We then cloned 14 pools of sgRNAs into a lenti-sgRNA hygro vector using Golden Gate sites or Gibson assembly [21, 68]. These sgRNAs targeted the ABL kinase domain (amino acids 242–320), aligning with the DMS region. To prevent overlapping edits and ensure individual sgRNA assignment within each pool, we staggered each sgRNA. In total, 71 ABL sgRNAs were evaluated, with five negative control sgRNAs (targeting mROSA26) spiked into each pool at ∼12%. The lentivirus library of sgRNAs was made by lipofection of HEK293T in 10-cm dishes as above. We performed three independent infections using 1.5 million Ba/F3 BCR-ABL WT ABE8e cells. Cells were cultured for 3 days before initiating 1 mg/ml hygromycin selection. After 6 days, hygromycin and IL-3 were withdrawn, and a cell pellet was collected. This initial pellet was used for both sgRNA and edit sequencing. Nine days post-IL-3 withdrawal, further cell pellets were harvested for both sgRNA and edit sequencing. Guide sequencing was performed as described above [21]. Paired end 300 × 300 edit sequencing was carried out using combinatorial dual-index next-generation sequencing primers (Supplementary data S1) on NextSeq 2000 using P1 XLEAP-SBS reagents. To mitigate the risk of index hopping, we incorporated a randomized stagger, ranging from 0 to 7 nucleotides, upstream of the primer binding site. This design ensures that each unique index combination corresponds to a distinct read start and end location. One microgram of genomic DNA was used for both sgRNA and edit sequencing. To prepare the sample for edit sequencing, the target region was amplified with high-fidelity UltraII Q5 polymerase (NEB M0544L) according to the following PCR protocol (Table 1).

**Table 1.** Polymerase chain reaction protocol for edit amplification

Temperature (°C)	Time	Cycles
95	2 min	1
95	10 s	
58	10 s	25
70	5 s	
65	5 min	1

A custom Python script was used to determine the frequency of in-phase or *cis* mutations made by ABE. Briefly, reads were trimmed and aligned to ABL cDNA using Bowtie 2 [69]. A mismatch was classified as a bona fide edit only if it possessed a quality score exceeding 20 in both the forward and reverse sequencing reads. Background mutation frequency was set based on the triplicate mean of the 95th percentile of mutant frequency in lentivirus-integrated BCR-ABL from a negative control library containing only mROSA26-targeting sgRNAs. We used an sgRNA frequency cutoff of 1% to identify high-confidence sgRNAs. Fold change of sgRNAs and edits was determined by pyDESeq2 [67]. Guide and edit growth rates were calculated by normalizing them against the known *WT* BCR-ABL Ba/F3 growth rate of 0.055 h^−1^. Specifically, the WT growth rate for guide analysis was derived from mROS26 spike-in data, while for edit analysis, it was based on reads containing no mismatches. All A > G mutations within the editing window of an sgRNA were linked to that sgRNA.

### BE-HIVE weighted model

In order to determine how well the on-target editing sgRNA phenotype can be recapitulated, sgRNA and editing efficiency was predicted by BE-HIVE [70]. Only edits with a frequency >0.05 and sgRNAs with completely matched DMS measurement were allowed. We model the growth rate of an sgRNA as the weighted sum of the growth rates of edited and unedited cells:


(2) \begin{eqnarray*}
{\rm sgRNA}\;{\rm growth}\;{\rm rate} &=& \left( {{\rm edit}\;{\rm proportion} \times {\rm edit}\;{\rm growth}\;{\rm rate}} \right)\nonumber\\ &&+\, \left( {{\rm unedited}\;{\rm proportion} \times 0.055} \right)
\end{eqnarray*}

In the case of simultaneous edits in cis across (*n*) multiple amino acids, a null model of mutant interactions predicts the growth rate of the multi-amino acid mutant as the product of individual mutant phenotypes [phenotype is growth rates (*r_n_*) divided by the WT growth rate (0.055 h^−1^)] multiplied by the WT growth rate [71]:


(3) \begin{eqnarray*}
{\rm WT}\mathop \prod \limits_{i = 1}^n \frac{{{r_n}}}{{{\rm WT}}}
\end{eqnarray*}

In other words, under our null model if Mutant A confers a growth rate that is 0.7× the WT growth rate and Mutant B confers the same disadvantage of 0.7× the WT growth rate, then the compound AB mutant is expected to reduce the 0.7-fold growth rate by another 0.7-fold for an expected compound mutant growth rate of 0.49× the WT growth rate. If there were an epistatic interaction, the growth rates of the double mutant would differ from this expectation.

## Results and discussion

### DMS data are high quality and correlates with evolutionary conservation, secondary structure, and function

For our comparison experiments, we chose to use a fit-for-purpose cellular system, where the growth of the cell line, Ba/F3, depends upon the presence of IL-3 in the media until an activated tyrosine kinase is added. This system allows us to introduce the DMS library in the same genomic context as the target of the base editor screen. Heterologous expression of a tyrosine kinase cDNA integrated into the genome is advantageous with respect to our study design because all variants (DMS or BE) are evaluated using the same promoter. We use the BCR-ABL oncogene as the activated tyrosine kinase for several reasons: (i) It is an important oncogene that is of interest to applied and basic research groups. (ii) Its structure has been solved many times in many conformations [72–74]. (iii) There are years of experiments that can be drawn from to gain confidence in the resultant data [73, 75, 76].

**Figure 1.**
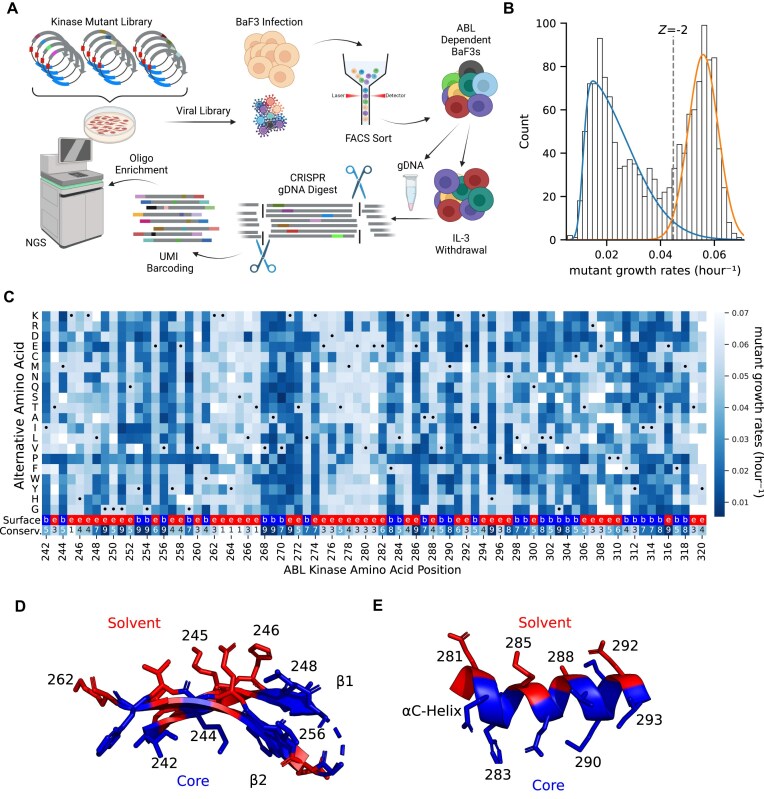
Functional landscape of ABL N-Lobe. (**A**) Schematic of DMS. After lentiviral integration of EGFP-P2A-BCR-ABL, Ba/F3s were sorted to enrich EGFP + infected cells. Cells were pelleted before and 6 days after IL-3 withdrawal. After targeted genomic DNA digest by Cas9, single molecules of DNA were labeled by UMI ligation. Then biotinylated oligo baits were used to enrich the mutagenized region. (**B**) The distribution of mutant growth rates in the ABL1 N-lobe is bimodal. Two skewed Gaussians are fit to determine the variation in deleterious (blue) and “WT-like” (orange) mutations. The dotted line represents a −2 Z-score threshold with respect to the “WT-like” distribution. (**C**) Heatmap of the growth rate of mutations at each position in ABL1 N-lobe. Black dot represents WT positions. Missing data are in white. Second to the last row of the heatmap provides surface exposure information because solvent exposed residues (red, “e”) tend to be more tolerant of mutations than buried residues (blue, “b”). The bottom row of the heatmap indicates the evolutionary conservation for each residue on a scale of 1 (low conservation) to 9 (high conservation). Tolerance/sensitivity to mutagenesis is projected onto two key structural features of the ABL1 N-lobe (PDB 6XR6): the (**D**) anti-parallel beta-sheet, and the (**E**) αC-Helix. If mean growth rate of alternative alleles at a residue is less than the −2 Z-score cutoff, then the residue is colored blue. In contrast, if the mean growth rate of alternative alleles is greater than the –2 Z-score cutoff, then it is colored in red. (*N* = 2).

To perform the DMS in BCR-ABL, we designed a saturation mutagenesis library spanning amino acid residues 242–320 in the N-lobe of the ABL1 kinase domain. This region was chosen for its reasonable size and the presence of known structural features that include the p-loop, the gatekeeper, and the alpha-C helix. This library was transduced into Ba/F3s in the presence of IL-3. After infection, FACS was used to enrich for infected cells. Following recovery from sorting, the Ba/F3 cells were screened for 6 days without IL3. In this negative selection screen, variants expressing non-functional copies of the BCR-ABL fusion protein deplete from the population. After the screen, we perform a sensitive barcoded sequencing protocol where the heterologous copy is specifically excised from the genome using Cas9-sgRNA complexes that are specific to the cDNA [56]. These genomic fragments are then ligated with unique molecular identifiers to allow for the deconvolution of library variants and the elimination of PCR and sequencing noise (Fig. 1A) [57, 58]. Genomic sequencing confirmed that the cell population contained a library representing 97% of all possible single amino acid variants spanning ABL1 residues 242–320.

When a mutant in BCR-ABL impairs kinase function, then the host cell will exhibit a reduced growth rate. After sequencing, we found that mutant growth rates form a bimodal distribution of counts (Fig. 1B), consistent with measured distributions of fitness effects in other DMS screens [17, 18, 28]. One of the peaks in this bimodal distribution of growth rates centered at ∼0.02 h^−1^ and constituted the set of deleterious mutations, while the other distribution mode was centered at the known WT growth rate of 0.055 h^−1^ and harbored AAs that are “WT-like fitness” (Fig. 1B). To call the “hits” of specific amino acid variants that are required for growth, we use a Z-score cutoff of −2 for the “WT-like” distribution. This specific cutoff was selected to be consistent with prior literature in the sgRNA community [34, 35, 62, 77] and to draw a line that can be approximated in both studies using approximately analogous statistical and experimental criteria (Fig. 1B). Using this cutoff, we estimate that 56% of measured variants in the N-lobe of the ABL kinase impair kinase function.

Initial observations of the deleterious residues in the DMS dataset are consistent with prior knowledge. For instance, the catalytic lysine K271 is required for kinase activity and mutations at that position strongly deplete (measured growth rate averaged 0.019 h^−1^) (Fig. 1C) [74]. Moreover, the systematic insertion of prolines during DMS studies causes a discernible “proline band” in high quality studies [28, 78]. We clearly observe a proline band in our data (Fig. 1C). Beyond these critical depletion signals, a known flexible and non-conserved region in ABL1 (i.e. residues 262–267 between the β2 and β3 strands) did not deplete.

More systematically, functionally important residues are typically evolutionarily conserved and their mutagenesis tends to be deleterious in DMS studies [10, 18]. Consistent with this, there is a −0.74 correlation (*P* < .001) between the mean mutant growth rate at a residue and the evolutionary conservation scores of that residue from ConSurf [72, 79–81] (Fig. 1C, bottom row of heatmap, Supplementary Fig. S1). Moreover, from a biophysical perspective, solvent accessible residues tend to be more tolerant of mutagenesis and are less conserved [82]. We observe this trend in Fig. 1C and D, and Supplementary Fig. S1.

The ABL1 N-lobe is composed of conserved structural elements that include the alpha-helix called the αC-helix, the five-stranded antiparallel β-sheet, the GxGxxG motif of the P-loop, as well as several catalytically important residues. Investigation of these key structural features reveals patterns that are consistent with known structure-function relationships. The GxGxxG of the P-loop spans residues 249–254 and all three conserved glycines show strong depletion phenotypes. Additionally, the alternating banding pattern in anti-parallel β -strands 1 and 2 (residues 242–262, Fig. 1D) is explained by essential residues facing toward the substrate pocket (blue residues, Fig. 1D). Moreover, the αC-helix, dynamically transitions from its “in” to its “out” state during kinase activation. This highly conserved regulatory dynamic requires support from the underlying hydrophobic core. Thus, the residues that interact with the core of the protein show the expected pattern of intolerance (blue, Fig. 1E), while the outward-facing residues show tolerance to mutations (red, Fig. 1E). It is the connection to known structure, function, and conservation data that gives us high confidence in the validity of the DMS data as a gold standard for comparison with sgRNA data.

### An adenosine BE screen isolates functionally important domains and residues

**Figure 2.**
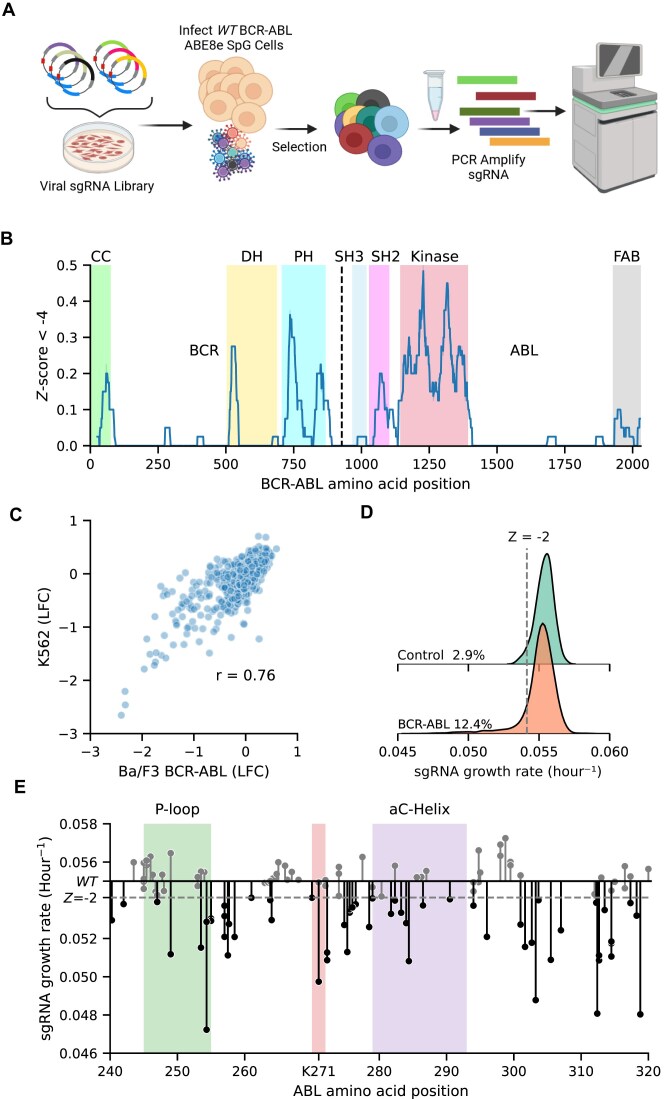
Adenosine base editor screen of full length of BCR-ABL. (**A**) Schematic of adenosine base editor screen. Three days after infection with BCR-ABL sgRNA library, Ba/F3 EGFP-P2A-BCR-ABL ABE8e cells were selected with 1 mg/ml hygromycin for 6 days and pelleted. Guides were PCR-amplified and sequenced. (**B**) A sliding window analysis using a window size of 40 sgRNAs. In a window we quantified the proportion of BCR-ABL sgRNAs that drop out more extreme than a Z-score of −4 of the negative control sgRNA growth rate (*N* = 3). (**C**) Correlation of the same ABE BCR-ABL screens performed in K562s (*N* = 2) and Ba/F3 expressing BCR-ABL (*N* = 3). (**D**) Kernel density estimate of growth rate distributions of non-targeting control, and BCR-ABL sgRNA libraries. Dashed gray line represents a –2 Z-score of the targeting control. (**E**) Lollipop plot displays dropout of each sgRNA across the ABL1 kinase domain. Dashed gray line represents a –2 Z-score of the targeting control (*N* = 3).

One of the major benefits of base editor (BE) screens is the ability to rapidly screen across a protein’s entire length. To demonstrate this breadth and speed, we rapidly cloned a tiled library of 3535 sgRNAs across the entire BCR-ABL cDNA, including regions beyond the original DMS library. The BE phenotype was determined by the change in sgRNA abundance following exponential growth in cell culture (Fig. 2A). We initially screened three different editors: ABE, CBE, and an nickase (nSpG) version of Cas9. While ABE had a high hit rate, CBE and nSpG had substantially lower hit rates (Table 2). Importantly, the CBE hits occur with sgRNAs that are expected to make edits in key residues, but both CBE and nSpG appeared limited in the breadth of phenotypes discovered. Based on this result, we focused our variant comparisons on our ABE data. Looking across the entire length of the protein (Fig. 2B), we performed a sliding window estimate of the proportion of strongly negative Z-scores across the protein with the ABE data (see “Materials and methods” section). This sliding window estimate highlights the regions of the protein that are unusually enriched for the presence of highly essential residues. Colored regions corresponding to the coiled-coil (CC) domain, double homology (DH) domain, plekstrin homology (PH) domain, Src-homology 3 (SH3), Src-homology 2 (SH2), the kinase domain, and F-actin binding domain (FAB) are highlighted. Highly significant domains that have been previously implicated in transformation, such as the CC [83, 84], SH2 [84–86], Kinase [87], and FAB domains [88, 89] show significant depletion. While the role of the DH domain is not understood, our base editor data suggests that it is important for growth factor independence (Fig. 2B) [90, 91].

**Table 2.** Hit rate among across different editors in Ba/F3 BCR-ABL screen

		Depleting guides	
Editor	Library	False	True
ABE	BCR-ABL	3488	47
	Control	1489	0
CBE	BCR-ABL	3531	4
	Control	1489	0
nSpG	BCR-ABL	3535	0
	Control	1489	0

Depleting guides are defined as guides with a log_2_ fold change of less than zero and an adjusted *P*-value of < .05.

While the matching genomic context of Ba/F3 (i.e. heterologous expression of BCR-ABL) for BE is useful for the comparison of BE and DMS, it is possible that editing variants in a Ba/F3 cell will yield a different result than editing variants in a K562 cell, which harbors true endogenous expression of BCR-ABL. To test for endogenous versus exogenous phenotypic differences, we also performed the same ABE screen in K562 cells, a human leukemia cell line, and we observed strongly correlated results with our Ba/F3 ABE screen (Pearson *r* = 0.76, Fig. 2C). Most of the spread in the points was due to sgRNAs that did not score in either screen, suggesting that noise in variants with no measured effects drove the correlation value.

Finally, to call depletion “hits” in our Ba/F3 screen we used a –2 Z-score based on the distribution of negative-control guides to be consistent with Hannah *et al.* [34] (Fig. 2C). Applying this criteria yields 387 hits, or ∼12.4% of all BCR-ABL sgRNAs (Fig. 2D), consistent with others [34, 62]. Most notably, there is a strong over-representation of depletion phenotypes for guides that target the ABL1 kinase domain, with 38% of kinase domain guides depleting below a –2 Z-score. In concordance with the DMS screen above, the guides that can target the conserved regions of the P-loop, Lys 271, and the buried region of the αC-helix produce deleterious phenotypes (Fig. 2E).

### Side-by-side comparison of ABE predicted edits and DMS screens

To assess the correlation between our ABE screen and our DMS, we compared growth rates of each individual sgRNA (from the ABE screen) to the growth rates of the variants that sgRNA was predicted to create (from the DMS). We focused on 80/118 sgRNAs for which all predicted edits have variant information in the DMS screen. (Figs 1C and 2E).

**Figure 3.**
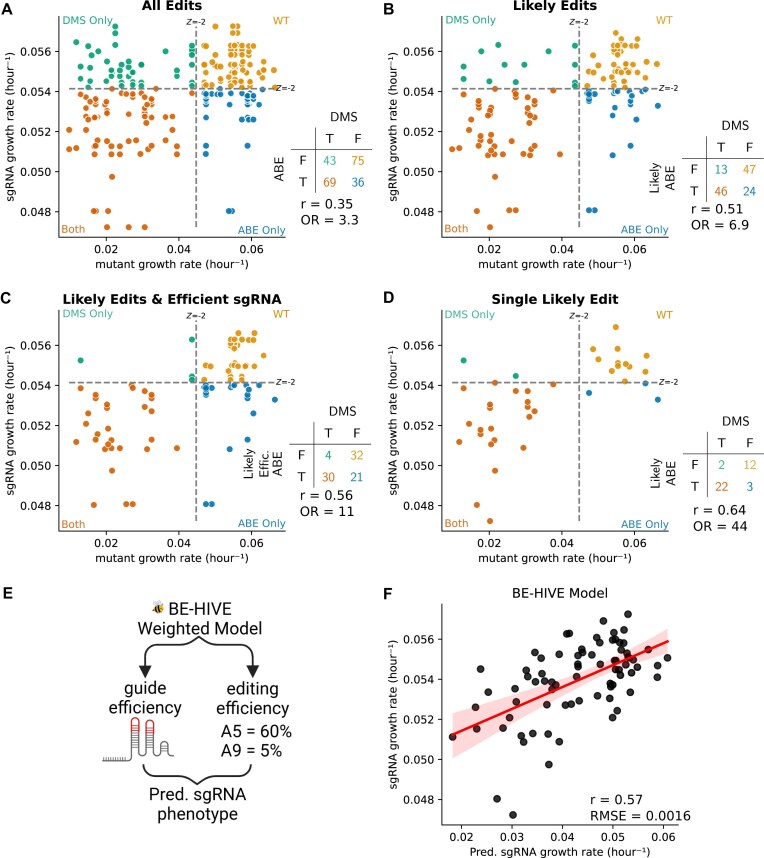
Comparison of adenosine base editor sgRNA growth rate and their respective mutation growth rates from DMS. Each dot represents a mutation an sgRNA is predicted to make. Dashed lines represent –2 Z-score of the non-deleterious distribution, and negative control sgRNA for the DMS and ABE screens, respectively. These cutoffs are used to define if an sgRNA or mutation is deleterious. If an sgRNA and its mutation do not deplete in their respective screen, in other words, both are non-deleterious, then they are colored yellow. If they both are deleterious, or true positive, then they are colored orange. If an sgRNA depletes, but the predicted edit does not deplete, a false positive, then the dot is colored in the blue. If a sgRNA fails to deplete, and the predicted mutation(s) are deleterious, a false negative, then that point is colored in green. (**A**) Shows all possible edits between nucleotides 2 and 12. (**B**) Shows only the most likely edits, those between nucleotides 4 and 8. (**C**) Shows only sgRNA predicted to be efficient, and edits between nucleotides 4 and 8. (**D**) Shows sgRNAs that are predicted to make only a single edit between nucleotides 4 and 8. (**E**) The distribution of edits can be estimated by machine learning model called BE-HIVE. (**F**) Correlation between predicted sgRNA growth rate and observed sgRNA growth rate. The *x*-axis shows the predicted growth rate of each sgRNA based on a weighted sum of the probability edit(s), and the effect of that edit(s) from DMS data. The *y*-axis shows the measured growth rate of the efficiently editing sgRNAs from the ABE screen.

The predicted edits of the sgRNAs predominantly occur within an editing window [43, 92]. While the editing window is in the non-targeting strand, we (and others) refer to the window relative to the guide sequence position. Most edits occur between positions 2 and 12 [92, 93]. Plotting sgRNA data against DMS data (Fig. 3A), each dot represents an individual variant, with each sgRNA appearing as a row of dots for all of its possible edits. An explanation of each quadrant is available in Supplementary Fig. S2. Notably, the dynamic range of the *Y*-axis is reduced for BE compared to DMS (there is an ∼0.045–0.06 h^−1^*Y*-axis measurement range for BE versus ∼0.01 –0.06 h^−1^ for DMS on the *X*-axis). This is likely because counting an sgRNA read can count either edited or unedited cells.

This initial analysis yields a modest but significant Pearson correlation of 0.35 (Fig. 3A). This indicates a relationship but suggests caution in directly annotating individual variants from a raw sgRNA experiment without further validation of the variants with individual sgRNAs. This view is in line with the current practice in the field.

Considering the modest correlation with all putative variants, we hypothesized that the 2–12 bp editing window was too broad. By focusing on a narrower, more efficient 4–8 nucleotide editing window [34, 93, 94], the Pearson correlation improved from 0.35 to 0.51, and the odds ratio (OR) of hits versus non-hits increased to 6.9 (Fig. 3B). This improved agreement came at the cost of removing 25 potentially correctly annotated variants (Fig. 3A and B, and Supplementary Fig. S3).

After applying the “likely” edits filter to generate Fig. 3B, we still identify 13 false negative and 24 false positive variants in our plots. In terms of these remaining false negatives, an sgRNA could fail to deplete when sgRNA sequences are of low efficiency [21]. Thus, we examined the consequences of adding an additional filter requiring an sgRNA efficiency score >50 (Fig. 3C and Supplementary Fig. S4) [21]. This simple additional cutoff further improves the Pearson correlation between sgRNA predicted edit growth rates and DMS data to 0.56, and eliminates 9 of the 13 remaining false negative variants (Supplementary Fig. S3).

Next, focusing on the false positive variants in Fig. 3B (blue dots), we identified 15 sgRNAs that are predicted to make 24 false positive variants. Interestingly, 10 of the 15 sgRNAs are multi-edit sgRNAs that are predicted to also make true positives edits (dark orange variant dots). One simple way to reduce the effect of multi-edit ambiguity is to only examine the sgRNAs that are predicted to make a single edit in the 4–8 nucleotide editing region. The addition of this “single likely edit” filter enhances the Pearson correlation to 0.64 (*P*-value < .001) and the OR for hits to 44 (*P*-value < .001) (Fig. 3D). For single-likely-edit sgRNAs there is a true positive rate of 0.88 and an accuracy of 0.87 with respect to gold standard DMS data. However, this filter removed 15 out of 40 original sgRNA hits, i.e. the vastly increased specificity comes at a 38% loss in sensitivity. For detailed information on filters and filtered sgRNAs/variants, see Supplementary Figs S3 and S4.

In order to expand the comparison to another cell line that expresses BCR-ABL from an endogenous genomic context, we performed the same comparison as in Fig. 3B and D, but we compared our DMS data in Ba/F3 to our ABE BE data in K562 cells. Interestingly, applying the same editing window filters from Fig. 3 to the K562 data yielded similar increases in Pearson correlation (Supplementary Fig. S5). This finding suggests that changing the genomic context and cell line does not dramatically change the results of this loss-of-function screen. It also suggests that the difference in the technique is a larger source of variation than the difference in genomic context.

Given that filtering sgRNAs by efficiency, edit probability, and the number of edits improves annotation confidence at the expense of total hits, we sought to utilize data more effectively from multi-edit sgRNAs (Fig. 3D). At a first pass, it seems like one might create variant-level interpretations for multi-edit sgRNA hits just by predicting the ensemble of sgRNA edits and their abundance in the population using machine learning algorithms like BE-HIVE [70]. However, while BE-HIVE can predict the ensemble of mutations (Fig. 3E), it can not predict the proportion of the measured phenotype that is attributable to those mutations. Therefore, we investigated whether multi-edit sgRNA dynamics could be predicted from the combined dynamics of their polyclonal edits. We used the BE-HIVE predicted allele frequencies vector (to approximate the structure of the polyclonal population), and simply assumed that all of the predicted variants grow according to the gold standard DMS growth rates. This approach estimated the expected multi-edit sgRNA growth rate as a function of the individual edits (Equation 3). This also provides insights into the contribution of in-window editing versus off-target effects to sgRNA dynamics. We observed a moderate Pearson correlation of 0.57 (*P*-value < .001) between the predicted and actual sgRNA growth rates (Fig. 3F). This suggests on-target editing is a major contributor to sgRNA growth rates. It also supports the feasibility of variant validation experiments with pooled sgRNAs by directly sequencing edited variants from genomic DNA. We will test this hypothesis in the next section.

### Pools of multi-edit sgRNAs create a pool of edited variants that can be measured by error-corrected deep sequencing of genomic DNA

**Figure 4.**
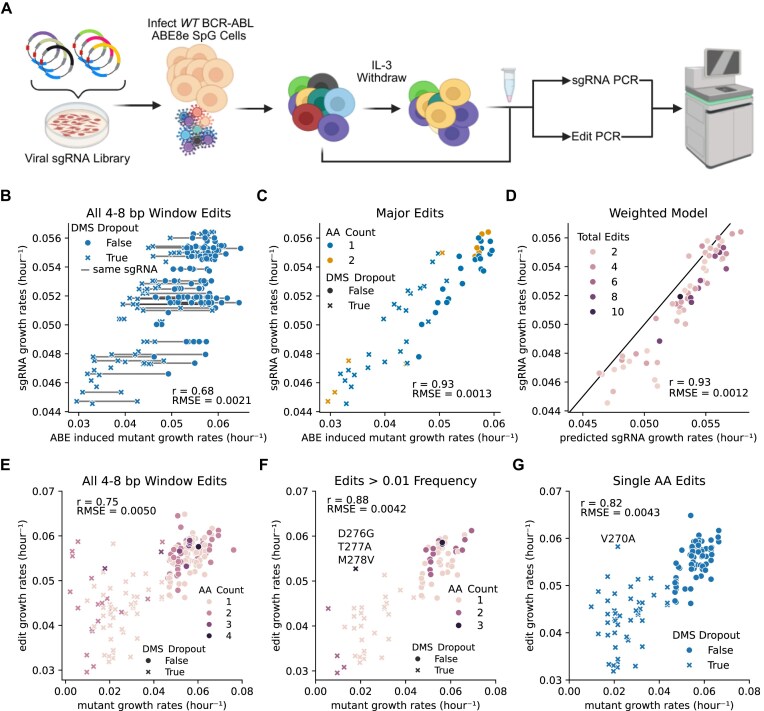
Medium-throughput pooled adenosine base editor screen. (**A**) Schematic of medium-scale validations screen of 71 sgRNAs targeting ABL1 kinase, where the edits and sgRNA are sequenced after IL-3 withdrawal. (B, C) Growth rates of sgRNA-induced edits. Each dot represents a specific edit and its measured growth rate, while each “X” or filled circle indicates whether the corresponding mutation was deleterious or nondeleterious in a prior DMS experiment. (**B**) Growth rates for all detected edits within the 4–8 nucleotide editing window of their respective sgRNAs. Gray lines connect edits generated by the same sgRNA. (**C**) Highlights the most prevalent edits, defined as those occurring at a frequency of over 50% of all edits within the sgRNA’s editing window. (**D**) Weighted model for sgRNA growth rate. The sgRNA growth rate was predicted by a weighted sum of the growth rates of all edits within its 4–8 bp editing window, with weights corresponding to the frequency of each edit. The black line indicates a perfect correlation between the predicted and experimentally measured sgRNA growth rates. (E–G) Comparison of growth rates from the pooled ABE screen and DMS data. These panels directly compare the growth rates of specific amino acid mutants measured in the pooled ABE screen and the prior DMS experiment. The amino acid (AA) count represents the number of amino acids simultaneously edited. (**E**) All edits detected within the 4–8 bp editing window. (**F**) Highlights high-confidence growth rate measurements by applying a stringent edit frequency cutoff of 0.01, and (**G**) focuses exclusively on single amino acid edits to enable a direct comparison between the ABE screen and DMS data (*N* = 3).

Our BE-HIVE analysis suggested that in-window editing drives multi-edit sgRNA growth rates (Fig. 3F). Therefore, we hypothesized that variant validation could be accelerated by directly sequencing variants in genomic DNA during pooled experiments. While doing this in a larger pool with thousands of sgRNAs is challenging because of the error rates involved in variant detection, sequencing the genomic DNA in pools with ∼100 sgRNAs is approachable with simple error correction schemes like TileSeq [54, 55]. This can be compared to the standard validation approach where sgRNA-variant relationships are tested in a one-by-one manner. The proposed approach involves medium-throughput validation pools of sgRNAs, and measuring the resultant variants directly in gDNA via error-corrected sequencing of the editing target. To test this, we selected 71 sgRNAs with varying predicted fitness measurements, aiming to determine if a variant is deleterious, and to explain sgRNA measurements through direct sequencing of the polyclonal pool (Fig. 4A). Sixty-three of the 71 selected sgRNAs made edits that were observable above the background mutation rate.

The measured sgRNA growth rates strongly correlated (Pearson *r* = 0.87, *P* < .001) with the sgRNA growth rates measured in the high-throughput screen from Fig. 2, confirming that the medium-throughput validation pool behaves similarly to the larger pool (Supplementary Fig. S6A). Sequencing the edits for each sgRNA revealed a wide range in the number of edits associated with individual sgRNAs (Supplemental Fig. S6B and C). Further analysis revealed a high Pearson correlation (*r* = 0.68) between sgRNA growth rates and the directly measured mutant growth rates (Fig. 4B), directly confirming that the fitness effects of sgRNAs are due to on-target editing. This correlation rose to 0.93 when considering only the major edit (defined as an edit whose frequency accounts for over 50% of an sgRNA’s total edits) (Fig. 4C). Even when incorporating all edits occurring within the likely editing window (nucleotides 4–8) using the weighted model from BE-HIVE (Fig. 3F; Equation 3), the correlation remained at 0.93 (Fig. 4D). This strong correlation suggests that while an sgRNA can generate multiple edits, a large majority of its phenotype is explained by these major edits.

If we directly compare edits made by ABE at a permissive variant frequency cutoff of 0.00015, based on the 95th percentile of mutant frequency detected in the absence of ABL1 targeting sgRNAs, and the mutations by DMS, we observe a Pearson correlation of 0.75 (Fig. 4E). This indicated that the direct sequencing of edited variants in resequencing pools can approach the quality of gold standard DMS data. However, some false negative edits appear to persist. This means that we directly observe edits that fail to drop out despite DMS data indicating they should. One explanation is that these edits are sequencing errors. Applying a more stringent background mutation cutoff of 1% of the relevant reads can eliminate these low-confidence measurements, which in turn improves the Pearson correlation to 0.88. The sole remaining strong outlier is a triple mutant (D276G T277A M278A) (Fig. 4F). While our compound mutation model (Equation 3) [71] predicts this triple mutant should deplete because M278V is strongly deleterious per DMS data, it’s possible that the addition of D276G and T277A rescue M278V’s deleterious effects on kinase function. To avoid potential confounding epistatic effects of compound mutations we also directly compare single amino acids mutants only, which still maintains a strong Pearson correlation of 0.82 (Fig. 4G).

## Conclusion

Thousands of human cell lines are routinely used to understand gene function in diverse tissue types, genetic backgrounds, and disease states [95, 96]. To plan how to perform variant annotation in these diverse contexts, the mammalian cell biologist/molecular geneticist requires a comparison of the variant editing tools that, at present, appear most suited to tackling the experimental challenges presented by human cell line diversity. Briefly, while HDR is a tremendous method that has made a huge impact, its use has largely been confined to haploid cell lines. Prime editing, while historically inefficient, has seen recent breakthroughs [23, 25]. However, these breakthroughs have required MLH1 KO [24] and the careful preselection of epegRNA sequences, resulting in an experimental method with uncertain transferability in WT cell lines [26]. Supplementary Table S1 has a summary of current methods alongside their strengths and weaknesses, while acknowledging that the field is changing rapidly. To enable the use of variant annotation tools by a broader community of mammalian cell biologists and geneticists, we sought to focus our benchmarking on two sets of tools (cDNA–DMS and BE) that have been already used in many different mammalian cell lines for functional genomic screens [22, 28, 31, 32, 34, 35, 40, 41, 62], and therefore represent the tools that are likely to be usable by the broadest set of audiences and biological questions.

In conclusion, our study provides a comprehensive comparison of DMS and CRISPR-based BE for variant annotation. We demonstrate that while DMS offers unparalleled depth and structural resolution, BE screening provides a rapid, broad, and efficient alternative at the cost of mutation diversity. By analyzing both methods in the same cellular and genomic context, we achieved a surprisingly high degree of correlation between the two, despite their inherent methodological differences and the potential for off-target effects and elevated mutation rates in BE. This robustness can clearly be seen in Fig. 4 which shows that sgRNA enrichment or depletion is largely interpretable as the composite sum of on-target edits within an editing window (*r* = 0.93).

We show that incorporating filters for sgRNA editing efficiency and reducing multi-edit ambiguity enhances the correlation between BE and DMS data. Our findings further reveal that variant annotation can be achieved directly from sequenced sgRNAs in BE screens when focusing on sgRNAs with single predicted edits within a narrow and efficient editing window. This streamlines the annotation process, particularly for variants exhibiting strong phenotypes. It also means that these single-edit sgRNAs sequenced directly from a pool can immediately classify a specific variant as deleterious and would be expected to create a ∼13% false positive rate directly from high-throughput screens. We also present a rapid way to deconvolute medium-throughput pools with error-corrected deep sequencing. Because sgRNAs are challenging to interpret when they are predicted to make multiple edits in their core editing window, we propose a two-step validation workflow using error-corrected deep sequencing. Error correction is necessary in medium-throughput pools because even a 100 sgRNA pool with editing efficiencies between 10% and 50% will create variant frequencies below the limit of detection (0.1%–1%) of conventional pooled NGS [55]. Figure 4 suggests that sequencing the variants with error correction in medium-sized validation pools of sgRNAs improves the quantitative correlation between base-edited variant annotation and cDNA-based DMS. We suggest the following workflow for base editor dropout screens: (i) Do a large unbiased screen (thousands of sgRNAs) where sequencing the sgRNA is used to deconvolute the pool. (ii) For sgRNAs that make single edits in the core editing window, annotate the predicted variant function directly from the screen in step i. (iii) Do a second validation screen of only the hits from step i using pool sizes of ∼100 sgRNAs. Directly sequence all the edits in these pools using error corrected TileSeq [54]. (iv) Use the direct sequencing data to validate all multi-edit variants.

One of the closest comparison results to the present study is that of Hanna *et al.* [34], who compared saturating genome editing [12] (akin to HDR-based DMS, performed at the University of Washington) with BE in BRCA1 in HAP1 cells performed at the Broad Institute. They reported a modest Pearson correlation (*r* = 0.44) [34]. A second similar study performed a comparison between cDNA-based DMS and prime editing in TP53 in A549 cells treated with a small molecule that selects for TP53 loss-of-function mutations [26]. However, the Pearson correlation they observed in this TP53 study was extraordinarily poor, with the authors arguing that supraphysiological overexpression of the TP53 mutant in the cDNA-based DMS screen was the cause.

Our comparison and our findings are different from both of these prior results in different systems. In our tightly controlled dropout screens, we observe a higher correlation than Hannah *et al.* [34] when limiting our comparisons to the core editing window. When comparing our K562 and Ba/F3 BE results (in the absence of splicing altering mutations), we suggest that the high correlation suggests that endogenous genomic context plays a minor role in variant annotation in our system. Thus, not all overexpression systems may have the same downsides that were observed in the case of TP53 [26]. We speculate that the dominant negative effects of TP53 are behind the importance of genome context in the A549 system. Our findings also extended to gain-of-function experiments, where imatinib treatment of Ba/F3 BCR-ABL cells showed comparable improvements (Supplementary Fig. S7). This was contingent on incorporating filters for likely edits and restricting analysis to single nucleotide edits within the 4–8 bp core editing window.

However, for complex cases involving multi-edit sgRNAs and double mutations, direct sequencing of mutant pools offers a robust validation strategy. By directly measuring the variants generated by pooled sgRNAs, we confirmed that the fitness effects observed in BE screens are primarily due to on-target editing in the sgRNA editing window. This approach allows for accurate variant annotation even in challenging scenarios, while maintaining a higher throughput.

Overall, our study also suggests that because these tools are measuring the same underlying phenomena, the complementary strengths versus weaknesses of cDNA DMS and BE screening for variant annotation might be combined for maximum impact in human cell biology and genetics across large regions of proteins, or entire protein networks. BE libraries can be rapidly deployed in a range of cell lines for editing at the endogenous locus. DMS in mammalian cells can then give structural resolution and detail in proteins. By strategically combining these two powerful tools, researchers can achieve efficient variant characterization across the genome with BE and structural detail with DMS. This will accelerate our understanding of gene function, structure-function, and disease mechanisms in diverse human cell lines.

## Supplementary Material

gkaf738_Supplemental_Files

## Data Availability

All sequencing data used in this study can be examined at Short Read Archives BioProject PRJNA1218278. All code used to analyze base editor and DMS data is available on GitHub and Figshare with the DOIs 10.6084/m9.figshare.29497922, 10.6084/m9.figshare.29497925, 10.6084/m9.figshare.29525993 and 10.6084/m9.figshare.29565386.
