# Supplementary material for: A side-by-side comparison of variant function measurements using deep mutational scanning and base editing
Source: Nucleic Acids Res. 2025 Jul 31;53(14):gkaf738. doi: 10.1093/nar/gkaf738 (PMC12311789; doi:10.1093/nar/gkaf738)
Supplement: gkaf738_Supplemental_Files [file gkaf738_supplemental_files.zip › Supplementary Figure legends.docx]

**Supplementary Figure S1.** Structural and evolutionary conservation is captured by Deep Mutational Scanning screen**.** (**A**) ConSurf evolutionary conservation score versus mean mutant growth rates for each residue in the ABL N-lobe. Each residue is color coded based on whether it is exposed (orange) or buried (blue) based on PDB structure 6XR6. (**B**) Contingency table and fisher exact test statistics comparing residue solvent exposure and if the mean mutant growth rate is less than –2 z-score cutoff drawn from the distribution of WT-like mutant growth rates. (N=3)

**Supplementary Figure S2.** Classification of Adenosine Base Editor (ABE) sgRNAs based on Deep Mutational Scanning (DMS) growth rates. This explanatory graph illustrates the classification of ABE sgRNAs and their predicted edits against the growth rates of mutations identified by DMS. The x-axis, representing mutant growth rates from the DMS screen, serves as the gold standard for predicting the functional consequence of edits made by each sgRNA in the y-axis. Dashed lines at -2 Z-scores indicate the cutoff for classifying an sgRNA or mutation as deleterious to ABL kinase function. The quadrants define four outcomes: True Negative (sgRNA does not deplete, predicted edit not deleterious), False Positive (sgRNA depletes, predicted edit not deleterious), False Negative (sgRNA does not deplete, predicted edit deleterious), and True Positive (sgRNA depletes, predicted edit deleterious).

**Supplementary Figure S3.** Variant quality control and scoring flow chart. Excluded variants are boxed in red, and included variants are boxed in blue.

**Supplementary Figure S4.** sgRNA quality control and scoring flow chart. Excluded sgRNAs are boxed in red, and included sgRNA are boxed in blue.

**Supplementary Figure S5.** Concordance of K562 BCR-ABL Adenosine Base Editor (N=2) and Ba/F3 BCR-ABL Deep Mutational Screens (N=3). Each blue dot represents a sgRNA-edit pair. The three panels illustrate different editing windows: "All Edits" displays predicted edits within a 2 to 12 bp window relative to the sgRNA sequence, "Likely Edits" focuses on predicted edits within a 4 to 8 bp editing window, and "Single Likely Edits" further refines this to include sgRNAs with only single nucleotide edits within the 4 to 8 bp editing window. Pearson's r correlation coefficient is reported at the bottom of each scatter plot.

**Supplementary Figure S6.** Characterization of the Ba/F3 BCR-ABL Adenosine Base Editor (ABE) verification screen. This follow-up screen utilized a select set of 71 sgRNAs targeting the ABL kinase. (**A**) Shows the Pearson correlation between the sgRNA growth rates from the original full-length BCR-ABL tiling screen (comprising 3535 sgRNAs) and the growth rates of the same sgRNAs in the verification screen. The distribution of (**B**) edits per guide and (**C**) the number of amino acids detected above background are also presented. (N=3)

**Supplemental Figure S7.** Comparison of Ba/F3 BCR-ABL adenosine base editor and deep mutational screens. The screens were performed following treatment with 500-600 nM imatinib for 6 days. The Pearson correlation between the two screens increases from 0.26 to 0.40 when comparing edits within the "likely" editing window (positions 4 to 8 relative to the sgRNA). The strongest Pearson correlation is observed when comparing sgRNAs that introduce only a single nucleotide variant within this likely editing window. (N=3)
