# Supplementary material for: A side-by-side comparison of variant function measurements using deep mutational scanning and base editing
Source: Nucleic Acids Res. 2025 Jul 31;53(14):gkaf738. doi: 10.1093/nar/gkaf738 (PMC12311789; doi:10.1093/nar/gkaf738)

**Supplementary Table 1.** Summary description of functional genomics methods.

| *METHOD* | *ENDOGENOUS MUTATION* | *WHOLE GENOME SCALING* | *VARIANT DIVERSITY* | *GENOME EDITING EFFICIENCY* | *REQUIRED OPTIMIZATION* | *HUMAN UTILIZATION*[1] |
|---|---|---|---|---|---|---|
| **SATURATING GENOME EDITING** | **Yes** | **No** | **High** | **Highest**[1] | **Medium**[2] | **Low** |
| **PRIME EDITING** | **Yes** | **Yes** | **High** | **Medium*** | **High**[3–5] | **Yes** |
| **BASE EDITING** | **Yes** | **Yes** | **Low** | **High**[6] | **Low** | **Yes** |
| **DEEP MUTATIONAL SCANNING** | **No** | **No** | **High** | **N/A** | **Low** | **Yes** |

*While recent advances have significantly boosted prime editing efficiency[3,7], it has historically struggled[5] without optimized epegRNAs and cell line development.

## Supplementary References


1. Maes, S., Deploey, N., Peelman, F. & Eyckerman, S. Deep mutational scanning of proteins in mammalian cells. *Cell Rep. Methods* **3**, 100641 (2023).
2. Obolenski, S., Olvera-León, R., Sun, D., Adams, D. J. & Waters, A. J. Protocol for the functional evaluation of genetic variants using saturation genome editing. *STAR Protoc.* **6**, 103710 (2025).
3. Yan, J. *et al.* Improving prime editing with an endogenous small RNA-binding protein. *Nature* **628**, 639–647 (2024).
4. Chen, P. J. *et al.* Enhanced prime editing systems by manipulating cellular determinants of editing outcomes. *Cell* **184**, 5635-5652.e29 (2021).
5. Gould, S. I. *et al.* High-throughput evaluation of genetic variants with prime editing sensor libraries. *Nat. Biotechnol.* 1–15 (2024) doi:10.1038/s41587-024-02172-9.
6. Sánchez-Rivera, F. J. *et al.* Base editing sensor libraries for high-throughput engineering and functional analysis of cancer-associated single nucleotide variants. *Nat. Biotechnol.* **40**, 862–873 (2022).
7. Cirincione, A. *et al.* A benchmarked, high-efficiency prime editing platform for multiplexed dropout screening. *Nat. Methods* **22**, 92–101 (2025).

Supplementary Figure S1 Sokirniy & Inam

A

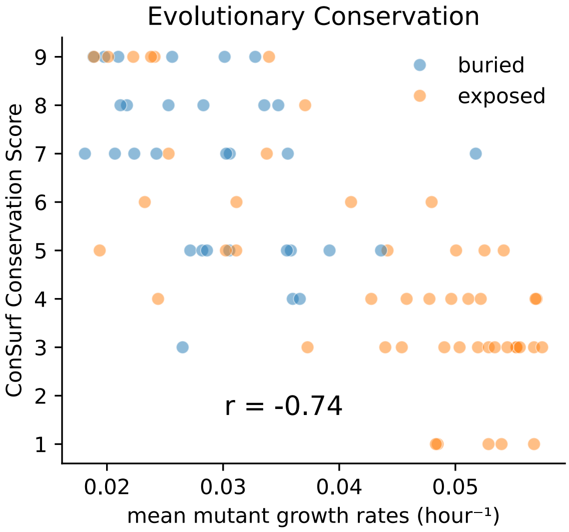


B

| Buried Residue | mean GR < -2 Z-score: T | mean GR < -2 Z-score: F |
|---|---|---|
| T | 30 | 1 |
| F | 20 | 28 |

OR = 42
$p < 0.001$

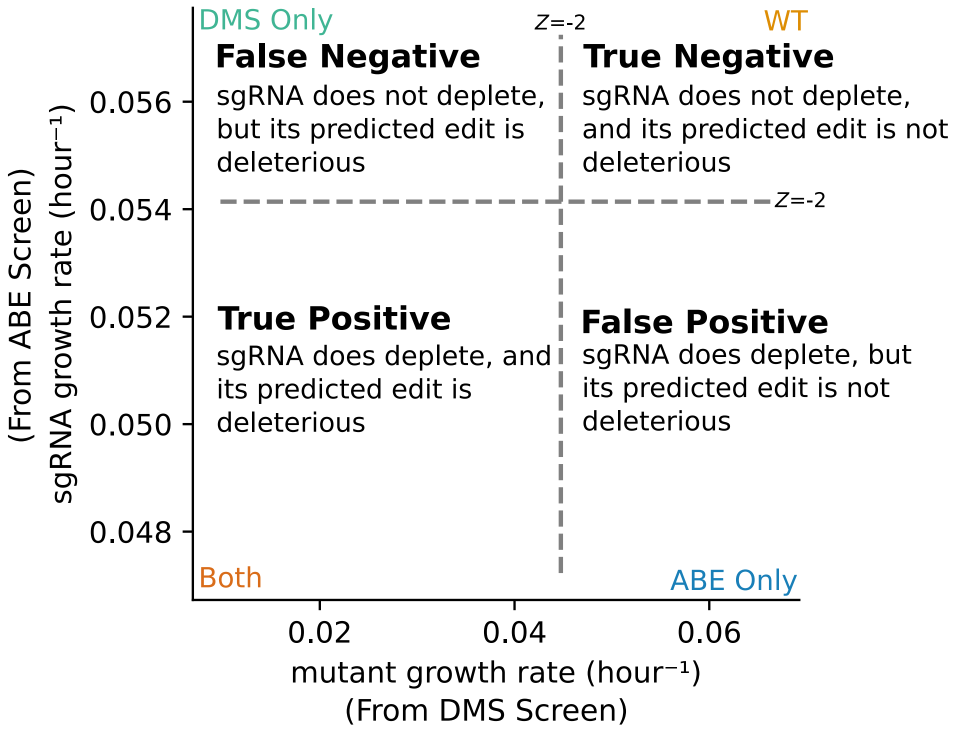
DMS Only
WT
Both
ABE Only
Z=-2
Z=-2
False Negative
sgRNA does not deplete, but its predicted edit is deleterious
True Negative
sgRNA does not deplete, and its predicted edit is not deleterious
True Positive
sgRNA does deplete, and its predicted edit is deleterious
False Positive
sgRNA does deplete, but its predicted edit is not deleterious
0.056
0.054
0.052
0.050
0.048
(From ABE Screen)
sgRNA growth rate (hour⁻¹)
0.02
0.04
0.06
mutant growth rate (hour⁻¹)
(From DMS Screen)

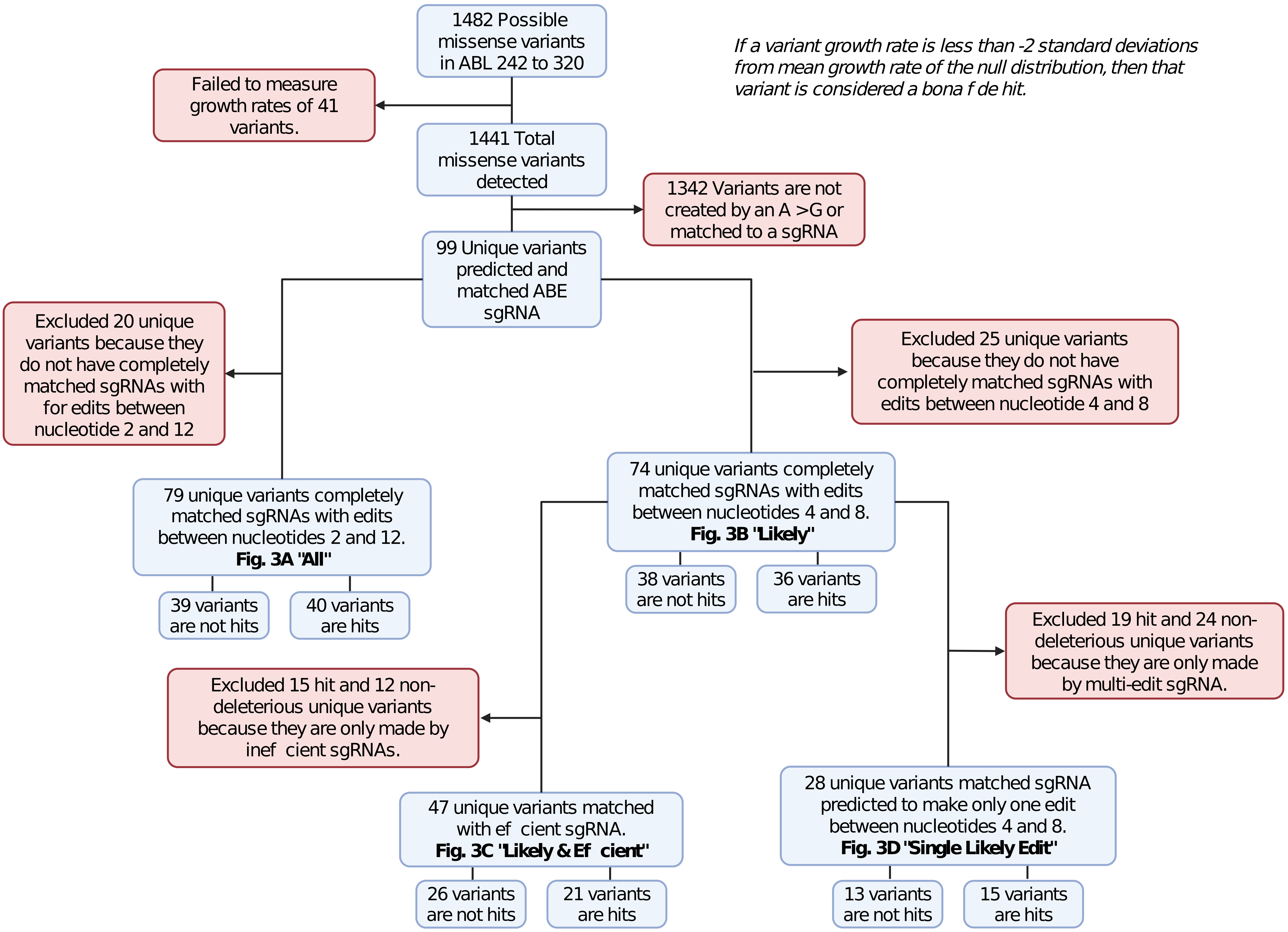
1482 Possible missense variants in ABL 242 to 320
If a variant growth rate is less than -2 standard deviations from mean growth rate of the null distribution, then that variant is considered a bona f de hit.
Failed to measure growth rates of 41 variants.
1441 Total missense variants detected
1342 Variants are not created by an A >G or matched to a sgRNA
99 Unique variants predicted and matched ABE sgRNA
Excluded 20 unique variants because they do not have completely matched sgRNAs with for edits between nucleotide 2 and 12
Excluded 25 unique variants because they do not have completely matched sgRNAs with edits between nucleotide 4 and 8
79 unique variants completely matched sgRNAs with edits between nucleotides 2 and 12. Fig. 3A "All"
39 variants are not hits
40 variants are hits
74 unique variants completely matched sgRNAs with edits between nucleotides 4 and 8. Fig. 3B "Likely"
38 variants are not hits
36 variants are hits
Excluded 19 hit and 24 non-deleterious unique variants because they are only made by multi-edit sgRNA.
Excluded 15 hit and 12 non-deleterious unique variants because they are only made by inef cient sgRNAs.
47 unique variants matched with ef cient sgRNA. Fig. 3C "Likely & Ef cient"
26 variants are not hits
21 variants are hits
28 unique variants matched sgRNA predicted to make only one edit between nucleotides 4 and 8. Fig. 3D "Single Likely Edit"
13 variants are not hits
15 variants are hits

Supplementary Figure S4 Sokirniy & Inam

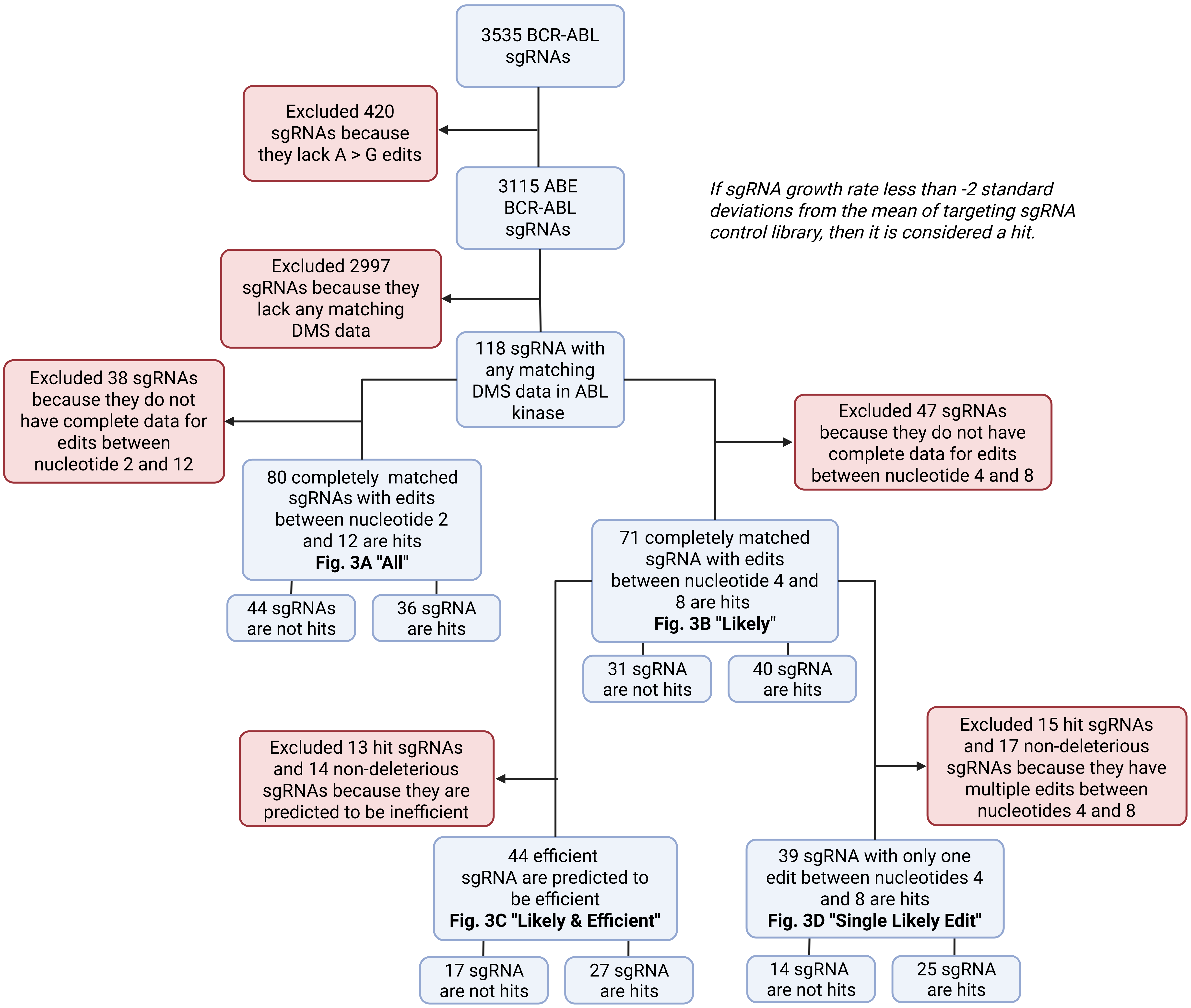

Supplementary Figure S5 Sokirniy & Inam

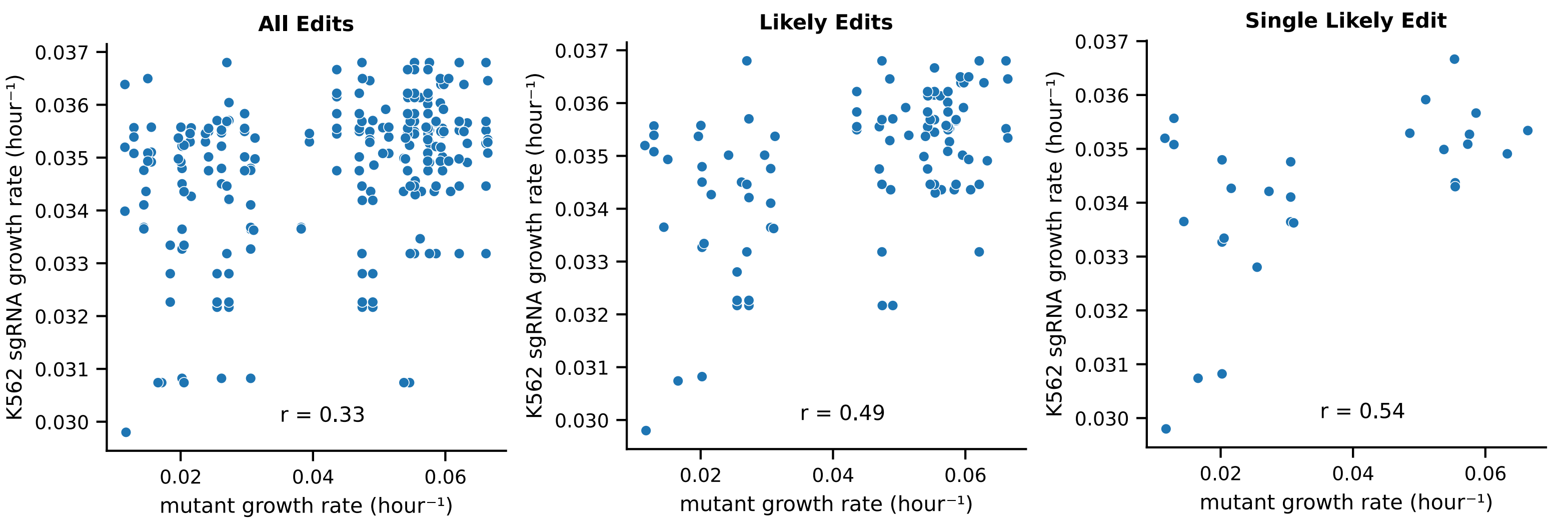

# Supplementary Figure S6

Sokirniy & Inam

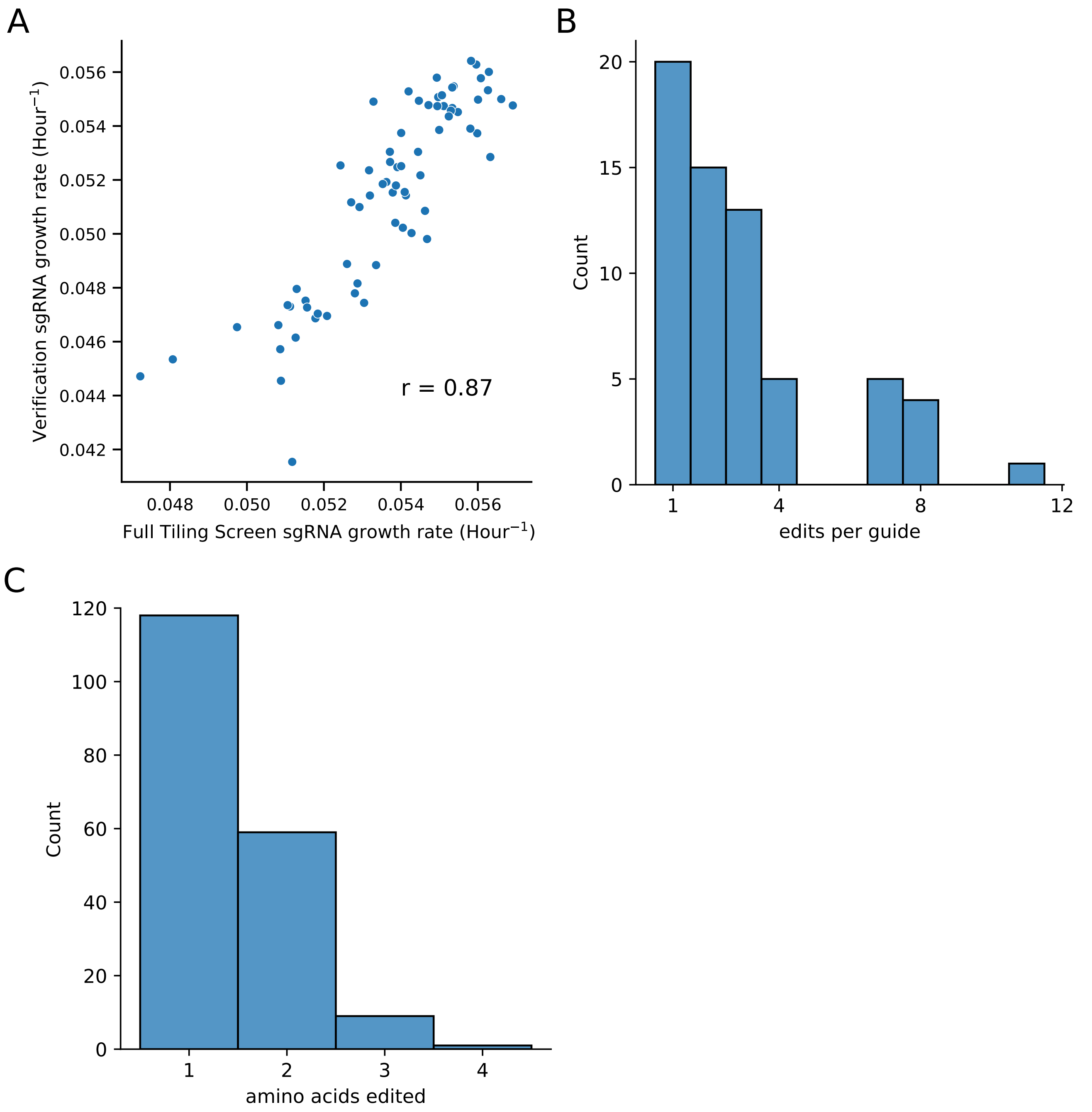

# Supplementary Figure S7 Sokirniy & Inam

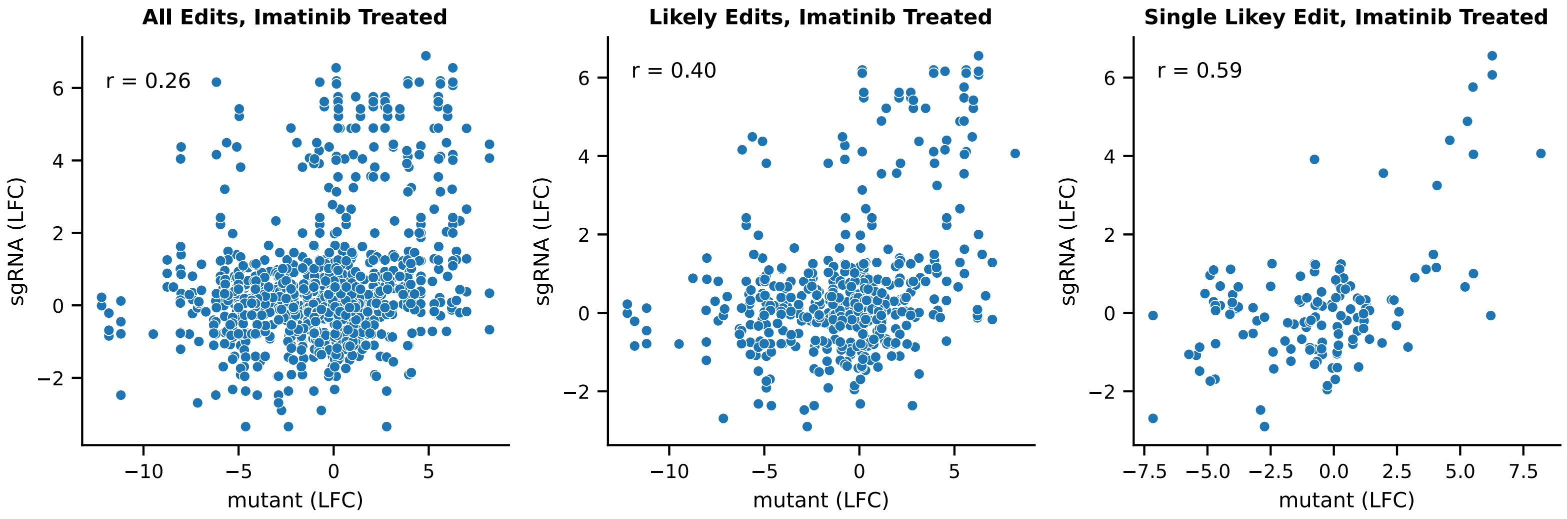

Supplement: gkaf738_Supplemental_Files [file gkaf738_supplemental_files.zip › Supplementary_Files_250701.pdf]
